# Diagnosis of HELLP Syndrome: A 10-Year Survey in a Perinatology Centre

**DOI:** 10.3390/ijerph16010109

**Published:** 2019-01-03

**Authors:** Kestutis Rimaitis, Lina Grauslyte, Asta Zavackiene, Vilda Baliuliene, Ruta Nadisauskiene, Andrius Macas

**Affiliations:** 1 Department of Anaesthesiogy, Lithuanian University of Health Sciences, Eivenių str. 2, LT-50009 Kaunas, Lithuania; lgrauslyte@gmail.com (L.G.); zavackieneasta@gmail.com (A.Z.); vilda.cesnovaite@gmail.com (V.B.); andrius.macas@kaunoklinikos.lt (A.M.); 2 Department of Obstetrics and Gynecology, Lithuanian University of Health Sciences, Eivenių str. 2, LT-50009 Kaunas, Lithuania; ruta.nadisauskiene@gmail.com

**Keywords:** HELLP syndrome, pregnancy-related hypertension, Mississippi protocol, diagnostic criteria

## Abstract

HELLP (Hemolysis, Elevated Liver enzymes, Low Platelet count) syndrome is a severe and rapidly progressing condition that requires distinct diagnostic considerations. The aim of this study was to evaluate the impact of the Mississippi triple-class system on the HELLP syndrome diagnosis, treatment, and outcomes in a perinatology centre during a 10-year period, and consider its effectiveness and necessity in everyday practice. A retrospective observational cohort study was carried out using the medical records of a tertiary perinatology centre with the diagnosis of HELLP syndrome from the period of time between 2005 and 2014. The patients who fit the HELLP syndrome diagnosis were grouped by the Mississippi triple-class system. The means of diagnosis and treatment outcomes within those groups were analysed statistically. There was insufficient statistical evidence of the blood pressure levels corresponding to the severity of patients’ condition (*p* > 0.05 in all of the groups). The clinical presentation varied within all of the classes, and the only objective means of diagnosis and evaluation of progression of the condition were laboratory tests. Even though HELLP syndrome is considered a hypertensive multi-organ disorder of pregnancy, the level of hypertension does not correlate to the severity of the condition; hence, the diagnosis should be based on biochemical laboratory evidence. Vigilance in suspicion and the recognition of HELLP syndrome and appropriate treatment are essential in order to ensure better maternal and neonatal outcomes.

## 1. Introduction

Hypertensive disorders of pregnancy complicate up to 10% of pregnancies and constitute one of the major causes of maternal and perinatal morbidity and mortality worldwide [1]. HELLP (Hemolysis, Elevated Liver enzymes, Low Platelet count) syndrome is a potentially life-threatening condition manifesting in the context of preeclampsia, which poses challenging diagnostic and management issues to the clinician [2]. At present, two major guidelines are used for HELLP syndrome diagnosis based on haemolysis, elevated liver enzymes, and low platelet (PLT) count, namely, the Tennessee classification and Mississippi triple-class system. According to the Mississippi triple-class system, the severity of HELLP syndrome is mostly characterised by the amount of PLT (Table 1) [3,4]. However, there is still a limited number of studies that would reveal the impact of the guideline application in everyday practice. There is also very limited observational data on the effect that the diagnostics and management approach has on the outcome on a larger scale. The aim of this study was to evaluate the impact of the Mississippi triple-class system on the HELLP syndrome diagnosis, treatment, and outcomes in a perinatology centre during a 10-year period, and consider its effectiveness and necessity in everyday practice.

## 2. Materials and Methods

This retrospective observational cohort study was carried out in a teaching hospital. No medical interventions were implemented during the study period, approval to access medical records was granted by the Lithuanian Medical Ethics commitee (No. BEC-MF-810). Medical records of all the women with the diagnosis of HELLP syndrome that were treated during the 10-year period between 2005 and 2014 were reviewed. Parturients were allocated to HELLP (HELLP Class 1, Class 2, and Class 3 subgroups), and partial HELLP groups based on Mississippi triple-class system diagnostic criteria. Cases that did not meet the HELLP nor partial HELLP syndrome criteria were excluded from the study. Investigation of the medical records was carried out by three experienced physicians, who work in an OB/GYN (obstetric/gynaecology) anaesthesiology and intensive care unit in the university hospital. Data were analysed statistically using IBM SPSS 20.0 package (IBM Corp., Armonk, NY, USA). The distribution for normality was evaluated by the Shapiro–Wilk test. Based on the results, the data was further assessed using the Kruskal–Wallis and Mann–Whitney U-tests (for the non-normally and normally distributed data, respectively). The significance level was set at 5% (*p* = 0.05).

## 3. Results

Between 2005 and 2014, there were 32,619 live births in our perinatology centre. Initially, according to medical records, a total of 64 patients were found to have been diagnosed with HELLP syndrome during the 10-year period. Following a detailed review of those records, 11 (17.19%) of parturients were excluded from the study, as they did not meet the criteria for HELLP nor partial HELLP syndrome diagnosis. The remaining 53 patients were included in the analysis, and 34 (65.4%) of them were classified as the HELLP group, whereas the remaining 19 (34.6%) fit the criteria for the partial HELLP syndrome group. All of the patients were Caucasian. The remaining demographic data and data regarding mode of delivery of all of the study parturients are presented in Table 2.

**Figure 1:**
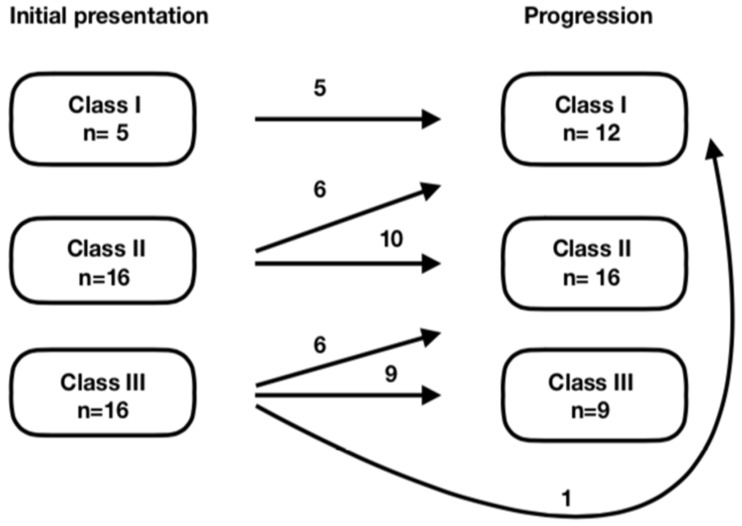
Initial presentation and progression of severity of the condition.

It should be noted that within the HELLP group on admission, based on the Mississippi triple-class system, five (14.7%) women fit the criteria of Class 1, 16 (47.1%) fit the criteria of Class 2, and 13 (38.2%) fit the criteria of Class 3 HELLP syndrome. In 13 cases, the patients’ symptoms and laboratory test results worsened, resulting in 12 (35.3%) patients with Class 1 and 16 (47.1%) with Class 2 HELLP syndrome (as shown in Figure 1).

**Figure 2:**
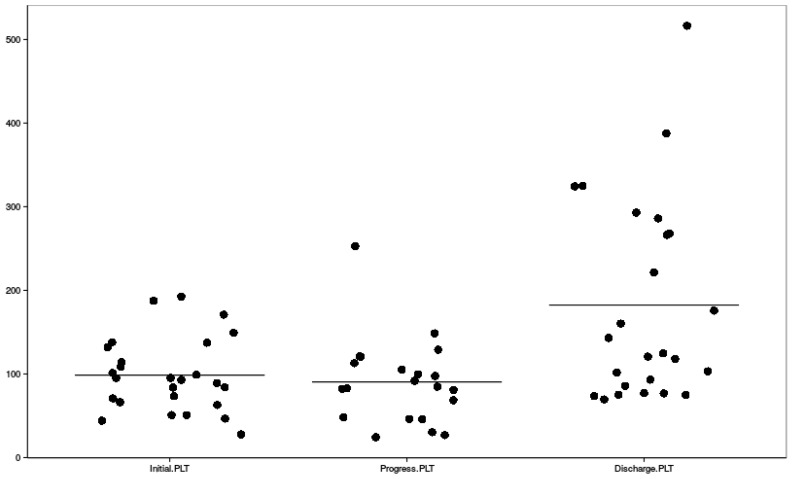
Dynamic changes of platelet count; PLT—platelet count.

**Figure 3:**
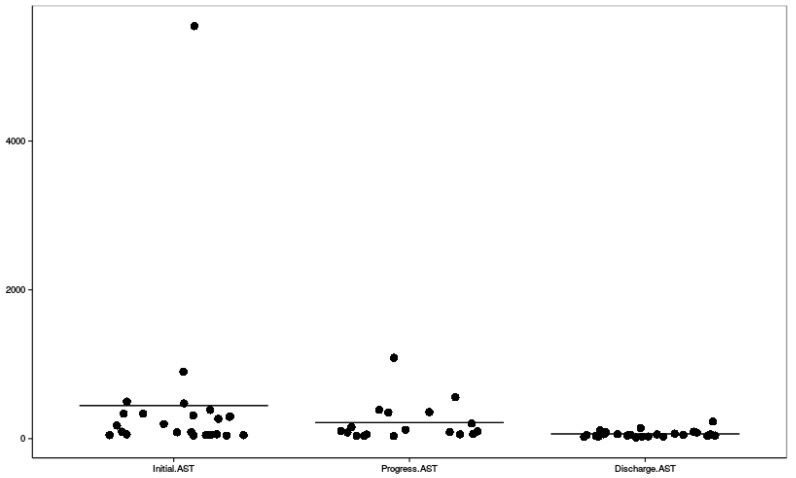
Dynamic changes of blood aspartate aminotransferase levels; AST—aspartate aminotransferase.

**Figure 4:**
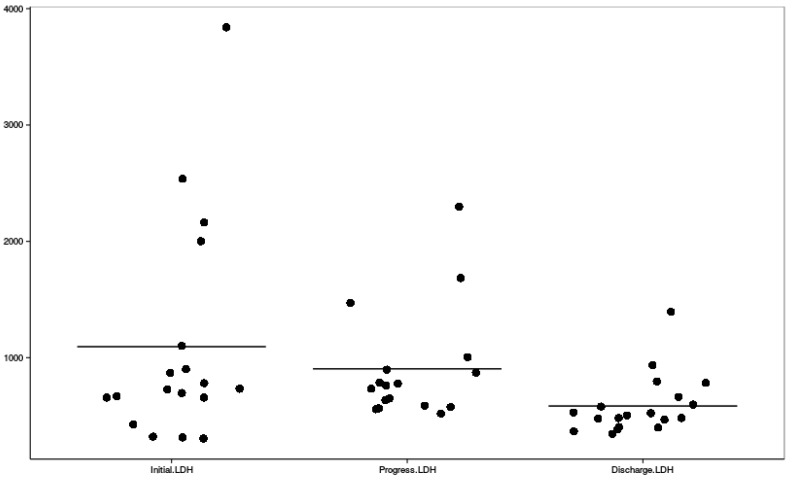
Dynamic changes of blood lactate dehydrogenase levels; LDH—lactate dehydrogenase.

Clinical symptoms, mean highest systolic and diastolic blood pressure measures, and their distribution among HELLP and partial HELLP groups are presented in Table 3, along with the treatment administered before delivery. Figure 2, Figure 3 and Figure 4 reflect the dynamic changes of the main laboratory tests. In some cases, data regarding laboratory values, repeat tests, and other diagnostic information was missing, which is apparent in the figures. Complications of all of the study group parturients are presented in Table 4.

The average diagnosis-to-delivery time in the HELLP group for patients who were admitted directly to our hospital or were transferred from other hospitals did not differ significantly, and was at 6.4 h (95% CI, 3.6 to 9.2, standard error of mean (SEM) = 1.41) and 6.8 h (95% CI, 3.8 to 10.6, SEM = 1.93), respectively. The average admittance-to-delivery time was 9.5 h (95% CI, 6.2 to 12.8, SEM = 1.67) for the HELLP syndrome patients who were admitted directly to our hospital, and 21.9 h (95% CI, 9.2 to 34.6, SEM = 6.5) for the transferred patients; however, the difference was not statistically significant. The average diagnosis-to-delivery and admittance-to-delivery time in the partial HELLP group had no significant differences based on the primary location of the patient. In all of the groups during this time, hypertension control, foetal lung maturation, and other measures aiming to prepare the mother and the foetus for delivery were taken.

## 4. Discussion

It is evident that the wide variety of possible clinical presentations of HELLP syndrome makes the clinical symptoms unreliable diagnostic criteria. Not only is it difficult to assess whether the severity of the patient’s condition can be predicted based on their subjective complaints, HELLP syndrome in its clinical presentation is similar to other conditions, such as benign thrombocytopenia of pregnancy, acute fatty liver of pregnancy, viral hepatitis, and others [5,6,7,8,9]. The symptoms of our study patients corresponded well with those described as most common in other studies [10,11,12,13]. It is of note that they did not in any way represent the severity of patients’ condition, and did not significantly influence the making of the final diagnosis, further highlighting the key role that laboratory tests play in the differential diagnosis of this condition. However, considering that there are certain tendencies in the clinical presentation of HELLP syndrome, all of the aforementioned symptoms should be noted in the diagnosis process, because they serve as “red flags” allowing clinicians to suspect HELLP syndrome in the first place.

The HELLP or partial HELLP syndrome diagnosis can be made during pregnancy or postpartum in women who were diagnosed with pregnancy-related hypertension (increase in the arterial blood pressure first detected after 20 weeks of gestation), with or without proteinuria. Despite many authors having shown that HELLP syndrome is a complication of preeclampsia or eclampsia, Sibai and Martin et al. observed that hypertension and proteinuria may be absent or only slight [14]. In our study, there was insufficient statistical evidence that the increased level of arterial blood pressure prior to delivery in all four groups (Class 1, Class 2, Class 3, and partial HELLP) correlates with the severity of HELLP syndrome (*p* > 0.05 in all groups). Hence, while arterial blood pressure is one of the main criteria in the diagnosis and treatment of preeclampsia, it is not as informative in the case of HELLP syndrome, and should not be used to predict its progression. It is important to recognise HELLP syndrome as a distinct entity that is associated with serious maternal morbidity [14].

Some authors have suggested intravenous dexamethasone as treatment to raise platelet counts and improve liver function test abnormalities [15]. The administration of intravenous corticosteroids antepartum remains a controversial approach to HELLP syndrome treatment. There are studies [16,17] that suggest that there is no benefit to the aforementioned treatment. However, the validity of these studies and their study design has been called into question by both Martin et al. and O’Brien [13,18]. Other studies have claimed that following the Mississippi protocol regarding intravenous dexamethasone administration allows clinicians to achieve four of the five goals: absence of maternal mortality, a reduction of major maternal morbidity (<20% in Class 1 and <5% in Class 2), a low rate of Class 3 or Class 2 HELLP syndrome progression to Class 1, and a mean length of hospitalisation following delivery of only four to 4.5 days [19]. However, there are no statistically and scientifically valid prospective randomised studies regarding this component of antepartum management due to ethical issues, rendering intravenous dexamethasone administration an individual medical institution protocol decision. In our hospital, since 2012, in the majority of cases, IV dexamethasone has been used in regard to thrombocytopenia as well as foetal lung maturation. Therefore, it remains unclear which criteria in which cases were the determining factors in choosing or forgoing the treatment with IV dexamethasone during the whole 10-year period of the study. Hence, it is difficult to analyse the effectiveness of the treatment strategy before delivery and its impact on the final outcomes. Failure to have a standardised treatment protocol not only makes it difficult to provide urgent care in the case of HELLP syndrome, it also makes it virtually impossible to compare treatment results between different studies, therefore showing the necessity of the implementation of both diagnostic and treatment protocols and guidelines.

It is advised to arrange the delivery within 24–48 h after the diagnosis is made [12,20]. Preeclampsia increases the cesarean rate, which ranges from 29.6% to 55.0%, with the percentage always being significantly higher than the incidence of cesareans among healthy pregnant women or pregnant women with isolated chronic hypertension [14]. In our hospital, in case of full-term pregnancy, the induction of vaginal delivery is the preferred approach, and in pre-term cases with an immature cervix, caesarean section is performed. In our study, 23 (67.6%) cases of HELLP syndrome ended in caesarean section. It should be taken into consideration that five women were diagnosed with placental abruption, while 23 (67.6%) of the deliveries were pre-term, and the majority of the patients had severe preeclampsia; therefore, it is difficult to determine whether the diagnosis of HELLP syndrome played the main role in choosing the route of delivery.

The occurrence rates of maternal and foetal HELLP syndrome complications in our study were consistent with those reported in other major studies [21,22,23]. Sadly, the data to assess the rate of acute kidney injury was not collected, as it seems to be associated with the rate of HELLP syndrome and have a correlation with further complications [24]. The perinatal mortality rate in our study was also within the ranges suggested by other studies [25,26,27,28,29]. There were no maternal deaths during our study period, once again proving that following the diagnostic protocol and treatment time, recommendations can help in reducing both perinatal and maternal complication and mortality rates.

Recently, an increasing number of articles have been published regarding the pathophysiology of HELLP syndrome, and there have been more and more studies aiming to find a laboratory marker that could possibly help diagnose HELLP syndrome early and objectively. There is research that provides promising new options such as the Elecsys immunoassay sFlt-1/PlGF (fms-like tyrosine kinase 1/placental growth factor) ratio [30] or using the modified Ham test [31]. However, it has to be kept in mind, that thus far none of the new options have been approved, as they still lack both sensitivity and specificity.

Certain difficulties arise when trying to compare clinical studies and reports regarding HELLP syndrome. Up until now, HELLP syndrome has had many different definitions, classifications, and criteria that have been used by fellow clinicians and scientists. This has limited the usefulness of many clinical reports. The Tennessee and Mississippi classifications are well-suited to facilitate comparisons. Classifications used in future reports should be restricted to one of these [24]. Using similar terms and being able to compare data should allow us to gain better understanding of the condition and in turn facilitate successful treatment.

## 5. Conclusions

HELLP syndrome is a potentially life-threatening dynamic condition for which a standardised approach to diagnosis and management is paramount. Even though HELLP syndrome is considered a hypertensive multi-organ disorder of pregnancy, the level of hypertension does not correlate to the severity of the condition; hence, the diagnosis should be based on biochemical laboratory evidence. It should be kept in mind that clinical presentation is one of the main factors that allow the timely diagnosis of this condition. Considering all of these factors, the implementation of standardised diagnostic criteria based on laboratory findings such as the Mississippi triple-class system for HELLP syndrome creates a possibility of defining this disorder similarly in most cases, applying the same treatment options in the same stage of the condition, and improving maternal and perinatal outcomes.

## Figures and Tables

**Table 1:** HELLP syndrome: Mississippi triple-class system [3].

HELLP Class	Mississippi Classification
1	PLT (μL) ≤ 50,000 AST or ALT ≥ 70 IU/L Total LDH ≥ 600 IU/L
2	50,000> PLT (μL) ≤ 100,000 AST or ALT ≥ 70 IU/L Total LDH ≥ 600 IU/L
3	100,000> PLT (μL) ≤ 150,000 AST or ALT ≥ 40 IU/L Total LDH ≥ 600 IU/L
Partial HELLP syndrome	Evidence of severe preeclampsia–eclampsia in association with two of three laboratory criteria for HELLP syndrome

HELLP: Hemolysis, Elevated Liver enzymes, Low Platelet count, PLT: low platelet, AST: aspartate aminotransferase, ALT: alanine aminotrasferase, LDH: lactate dehydrogenase.

**Table 2:** Demographic and data regarding delivery of all of the patient groups.

Variable	HELLP Class 1 *n* = 12 (35.3%)	HELLP Class 2 *n* = 16 (47.1%)	HELLP Class 3 *n* = 6 (17.6%)	Total HELLP *n* = 34	Partial HELLP *n* = 19
Age	28 ± 4.9	28 ± 5.9	29 ± 5.6	28 ± 5.6	32 ± 6.9
Primigravida	8 (66.7%)	10 (62.5%)	3 (50%)	21 (61.8%)	9 (47.4%)
Primipara	9 (75%)	11 (68.8%)	3 (50%)	23 (67.6%)	10 (52.6%)
Average gestational age at delivery	35 ± 4.0	32 ± 5.1	34 ± 4.6	33 ± 4.6	33 ± 3.9
Prior to the 27th week	1 (8.3%)	2 (12.5%)	0 (0%)	3 (8.8%)	2 (10.5%)
27th–37th	6 (50%)	9 (56.3%)	5 (83.3%)	20 (58.8%)	15 (78.9%)
After the 37th week	4 (33.3%)	3 (18.8%)	1 (16.7%)	8 (23.5%)	2 (10.5%)
Postpartum	1 (8.3%)	2 (12.5%)	0 (0%)	3 (8.8%)	0 (0%)
Severe preeclampsia	12 (100%)	15 (93.8%)	6 (100%)	33 (97.1%)	19 (100%)
Eclampsia	1 (8.3%)	1 (6.3%)	0 (0%)	2 (5.9%)	1 (5.3%)
Route of delivery:					
Vaginal	4 (33.3%)	5 (31.2%)	2 (33.3%)	11 (32.4%)	9 (47.4%)
Caesarean	8 (66.7%)	11 (68.8%)	4 (66.7%)	23 (67.6%)	10 (52.6%)
Anaesthesia technique:					
Spinal	1 (12.5%)	4 (36.4%)	2 (50%)	7 (30.4%)	2 (20%)
General	7 (87.5%)	7 (63.6%)	2 (50%)	16 (69.6%)	8 (80%)

**Table 3:** Distribution of clinical symptoms, mean arterial blood pressures before and after delivery, and treatment administered before delivery in HELLP and partial HELLP groups.

Variable	HELLP Class 1 *n* = 12 (35.3%)	HELLP Class 2 *n* = 16 (47.1%)	HELLP Class 3 *n* = 6 (17.6%)	Total HELLP *n* = 34	Partial HELLP *n* = 19	*p* Value
Symptoms:						
Headache	0 (0%)	5 (31.2%)	1 (16.7%)	6 (17.6%)	7 (36.8%)	0.037
Epigastric pain	8 (66.7%)	6 (37.5%)	3 (50%)	17 (50%)	8 (42.1%)	0.604
Generalised edema	3 (25%)	2 (12.5%)	0 (0%)	5 (14.7%)	4 (21.1%)	0.302
Nausea/vomiting	5 (41.7%)	5 (31.2%)	3 (50%)	13 (38.2%)	9 (21.1%)	0.965
Visual disturbances	1 (12.5%)	1 (6.3%)	1 (16.7%)	3 (8.8%)	2 (10.5%)	0.916
Highest BP before delivery, S/D	168 ± 21/ 105 ± 15	174 ± 34/ 105 ± 21	152 ± 23/ 97 ± 16	168 ± 28/ 104 ± 18	177 ± 31/ 101 ± 14	0.462/0.945
Highest BP after delivery, S/D	163 ± 13/ 97 ± 15	155 ± 26/ 97 ± 18	147 ± 22/ 85 ± 10	157 ± 21/ 96 ± 16	151 ± 25/ 90 ± 14	0.349/0.130
Antihypertensive medication	12 (100%)	16 (100%)	6 (100%)	34 (100%)	17 (89.5%)	
Magnesium sulfate	12 (100%)	15 (93.8%)	6 (100%)	33 (97.1%)	17 (89.5%)	
Corticosteroids	100% of cases with gestational age between 24 and 35 weeks					

BP—arterial blood pressure, S/D—systolic/diastolic.

**Table 4:** Prevalence of complications in all study groups.

Complication	HELLP Class 1 *n* = 12 (35.3%)	HELLP Class 2 *n* = 16 (47.1%)	HELLP Class 3 *n* = 6 (17.6%)	Total HELLP *n* = 34	Partial HELLP *n* = 19
Partial placental abruption	2 (16.7%)	1 (6.3%)	2 (33.3%)	5 (14.7%)	0 (0%)
Hepatic and renal failure	2 (16.7%)	1 (6.3%)	0 (0%)	3 (8.8%)	0 (0%)
Intracerebral hemorrhage	1 (8.3%)	0 (0%)	0 (0%)	1 (2.9%)	1 (5.3%)
Perinatal death	1 (8.3%)	3 (18.8%)	1 (16.7%)	5 (14.7%)	0 (0%)
